# Pneumocystis Pneumonia in a Non-Immunocompromised Lung Cancer Patient after Surgery: A Case Report

**DOI:** 10.3390/healthcare10102063

**Published:** 2022-10-17

**Authors:** Tae-Woo Kim, Jun-Ho Lee, Hyo-Jin Lee, So-Woon Kim, Hye-Sook Choi

**Affiliations:** 1 Department of Internal Medicine, Kyung Hee Medical Center, Seoul 02447, Korea; 2 Department of Pathology, Kyung Hee Medical Center, Seoul 02447, Korea

**Keywords:** Pneumocystis pneumonia, *Pneumocystis jirovecii*, non-immunocompromised, lung cancer, lobectomy

## Abstract

We present the Pneumocystis pneumonia case of a 64-year-old man with no remarkable history except for hypertension, who had not undergone any treatment other than surgery. On postoperative day 7, high-resolution computed tomography findings revealed multifocal ground-glass opacifications with interlobular septal thickening in both lungs; therefore, atypical pneumonia was suspected. Polymerase chain reaction (PCR) test performed after bronchoalveolar lavage was positive for *Pneumocystis jirovecii (P. jirovecii)*. Based on the PCR results, a final diagnosis of Pneumocystis pneumonia (PCP) was made. After treatment, he improved and was discharged. This is a unique case of PCP diagnosis in a non-immunocompromised patient, with no remarkable history except for hypertension, who had not undergone any treatment other than surgery for cancer. Thus, it is necessary to consider additional risk factors for PCP and timing of preventive treatment.

## 1. Introduction

Pneumocystis pneumonia (PCP), caused by *Pneumocystis jirovecii (P. jirovecii)*, is a common opportunistic infection associated with high mortality rates [1,2]. Well-defined risk factors for PCP include immunocompromised status, human immunodeficiency virus (HIV) infection, organ transplantation, and hematological malignancies [3]. There are some case reports on the diagnosis and high mortality rate of PCP in immunocompromised patients [3,4,5]. However, herein, we report the case of a non-immunocompromised lung cancer patient who developed PCP after right upper lobe (RUL) lobectomy and mediastinal lymph node dissection (MLND).

## 2. Case Presentation

**Figure 1:**
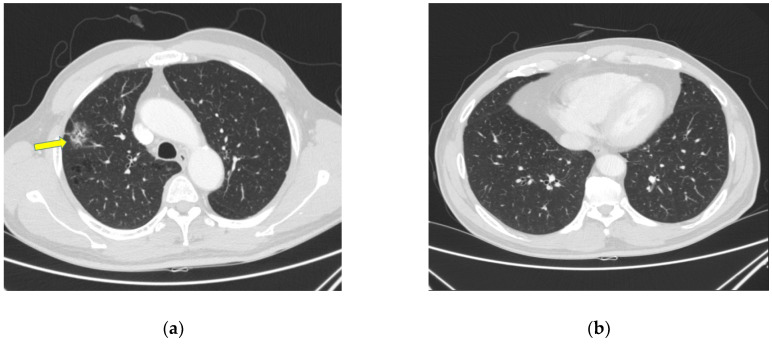
Images of initial chest computed tomography. (**a**) About 2 cm sized ground-glass opacification lesion suggesting malignancy indicated by arrow was on right upper lobe. (**b**) normal axial view.

A 64-year-old man underwent treatment at a local hospital for hypertension, and a ground-glass opacification (GGO) of approximately 2 cm, suggesting malignancy on the right upper lobe, was observed on initial chest computed tomography (CT) (Figure 1).

He was hospitalized for a percutaneous cutting needle biopsy (PCNB) after visiting the outpatient clinic of the Division of Pulmonary Medicine. He was an ex-smoker with a smoking history of 60 pack-years and had quit smoking 14 years ago. The PCNB conducted on 21 January 2020 revealed a non-small cell lung cancer (adenocarcinoma [ADC], stage IA2 [T1bNxMx]), but no distant metastasis was detected on additional imaging tests. He was discharged and re-hospitalized in the cardiothoracic surgery department on 17 February 2020, for surgical treatment of lung cancer.

His initial vital signs were normal, with a blood pressure of 133/86 mmHg, heart rate of 94 beats/min; respiratory rate of 20 breaths/min, oxygen saturation of 97% in room air, and body temperature of 36.3 °C. No abnormal sounds were observed during chest auscultation. Chest radiography revealed no abnormalities other than nodules in the RUL. Laboratory test results were within the normal ranges as follows: white blood cell (WBC) count, 7000/µL (neutrophils, 52.9%; lymphocytes, 36.5%; eosinophils, 4.4%); hemoglobin, 14.4 g/dL; and platelet count, 230,000/µL on complete blood and differential counts. Biochemical tests did not reveal any specific findings. The patient tested negative for HIV antibodies.

The patient’s surgical adequacy was evaluated through multidisciplinary treatment including an anesthesiologist. The patient underwent RUL lobectomy and MLND on 19 February 2020, confirming the pathological aspects of the ADC diagnosis, namely, a predominant lepidic pattern, well-differentiated, pT1bN0M0, and stage IA2. A one lung mechanical ventilator in pressure reserve volume control mode was applied during the operation. After the operation, it was confirmed that spontaneous respiration was possible and the vital signs were stable. Therefore, mechanical ventilation was stopped and the patient returned to the general ward. There was no fever postoperatively, however, inflammatory markers such as C-reactive protein and WBC count continued to increase. Antibiotics (piperacillin/tazobactam and levofloxacin) were administered in cooperation with the Division of Infectious Medicine.

**Figure 2:**
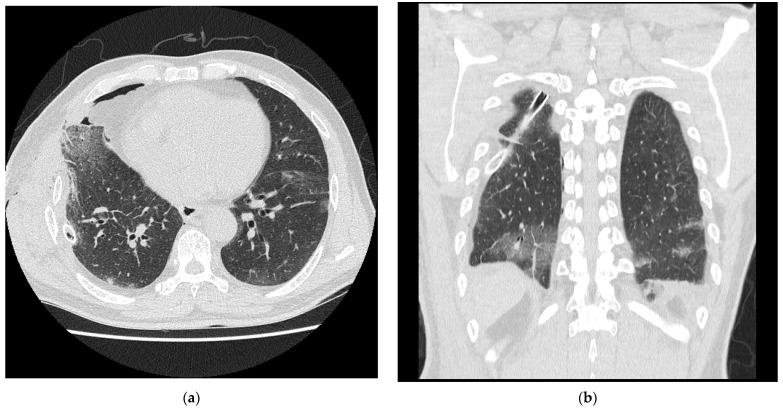
The high-resolution computed tomography revealed multifocal ground-glass opacifications with interlobular septal thickening in both the lungs. (**a**) Axial view, same position as (**b**), (**b**) Coronal view.

On postoperative day (POD) 7, a high-resolution CT (HRCT) was performed due to the deterioration observed on the chest radiography (Figure 2).

HRCT revealed multifocal GGOs with interlobular septal thickening in both lungs developed after surgery. The COVID-19 and *Mycoplasma pneumonia* PCR results were negative. On POD 14 (4 March 2020), the cough and dyspnea worsened, despite the use of antitussive agents and antibiotics. Saturation of peripheral O_2_ decreased from 97% to 93%, and new-onset dyspnea was also noted. Therefore, 2-L per minute (LPM) oxygen was supplied through nasal prong. Bronchoalveolar lavage (BAL) was performed on POD 15.

**Figure 3:**
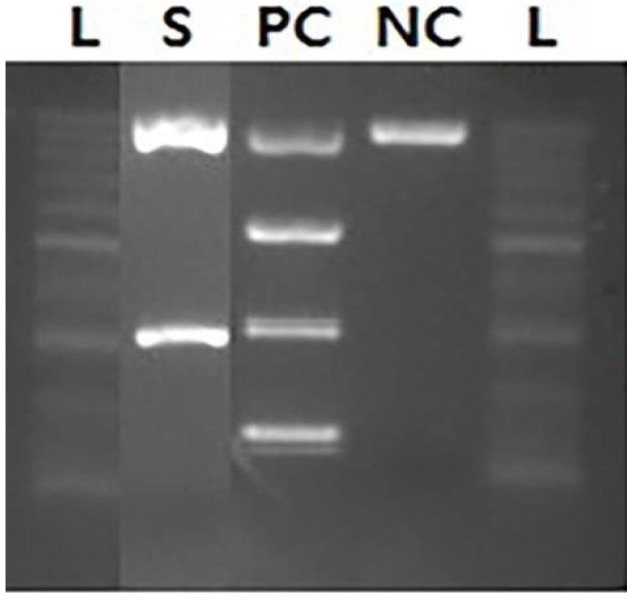
Electrophoresis results showing a positive for *Pneumocystis jirovecii*. The L column represents a 100 base pairs ladder. The S column shows the result of PCR sample. The PC and NC columns represent results for positive and negative controls, respectively.

The differential count of the BAL sample was as follows: WBC count, 695/µL (neutrophils, 15%; eosinophils, 8%; and macrophages, 68%), CD4 (57.2%) and CD8 (22.7%). Therefore, eosinophilic pneumonia and interstitial lung disease were excluded. Gram stain, acid-fast bacilli stain, cytomegalovirus real-time PCR, and Aspergillus antigen and culture tests were all negative. However, the 338 base pairs obtained after PCR for *P. jirovecii* were electrophoresed and confirmed to be positive (Figure 3); therefore, PCP was finally diagnosed.

**Figure 4:**
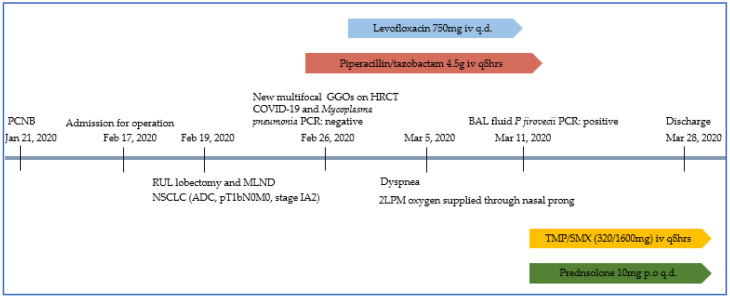
Synoptic flowchart containing patient information. PCNB = percutaneous cutting needle biopsy, RUL = right upper lobe, MLND= mediastinal lymph node dissection, NSCLC = non-small cell lung cacner, ADC = adenocarcinoma, HRCT = high-resolution computed tomography, GGO = ground-glass opacification, PCR = polymerase chain reaction, LPM = liter per minute, BAL = bronchoalveolar lavage, *P. jirovecii* = *Pneumocystis jirovecii*, TMP/SMX = Trimethoprim Sulfamethoxazole.

As per the treatment strategy for PCP, we discontinued the existing antibiotics and administered trimethoprim/sulfamethoxazole with steroid. Since the clinical features improved on POD 38 (28 March 2020), the patient was discharged from the hospital (Figure 4).

He has been continuously monitored in the outpatient department of the division of pulmonary medicine.

## 3. Discussion

Lung cancer is the most common cause of solid malignancy-related death, and infection and pneumonia are typically associated with poor prognosis [4,6]. In recent studies, the incidence of PCP in cases of solid tumors, including lung cancer, has been low (<25 cases per 100,000 patient-years). PCP is associated with radiotherapy, concurrent chemoradiotherapy, lymphopenia, and prolonged high-dose steroid therapy (20 mg of prednisolone equivalent per day for ≥3 weeks) [4,6].

PCP is a common opportunistic infection that is associated with high mortality rates. Mortality can be attributed to inactivated alveolar macrophages, which undergo apoptosis followed by the activation of caspase 9 present in the lungs and alveolar macrophages [1,2]. Currently, molecular assays, such as PCR test of respiratory samples (e.g., spontaneous sputum, induced sputum, BAL samples, or tissue samples), are available for direct DNA detection of the target organism [2]. Owing to the high mortality rate and invasive sampling, many studies have highlighted the importance of PCP prophylaxis and the exploration of effective diagnostic methods [2,5,7,8,9].

However, a positive PCR test is not indicative of PCP. The colonized microorganism must be distinguished from the cause of the disease [2]. Several studies have been conducted to identify PCR values that can distinguish PCP from colonization. Some studies have revealed that these values may be a positive predictive value (PPV) of up to 88.6% and negative predictive value (NPV) of 100% [10]. The criteria for colonization are based on negative microscopic *P. jirovecii* detection in pulmonary specimens and positive PCR results for *P. jirovecii* DNA detection [11]. PCP may be defined as definite, probable, possible, or not PCP, based on microscopic confirmation; DNA detection through PCR; clinical symptoms such as dyspnea, cough, and fever; and presence of bilateral perihilar lung interstitial infiltration and GGO on chest radiography or CT [1,2,6,8,11]. Based on these diagnostic criteria, the patient was diagnosed with probable PCP.

In previous studies, the clinicopathological characteristics of lung cancer patients diagnosed with PCP were chemoradiotherapy treatment, metastatic lung cancer, prior steroid treatment, and HIV infection [3,12,13,14].

This case is unique because PCP was diagnosed after surgical treatment for a newly diagnosed lung cancer in a patient with no remarkable history, except for hypertension. The patient was diagnosed with early stage NSCLC and had not undergone any treatment other than surgical treatment for cancer. There were no other signs of infection and specific findings on blood tests in the patient. Although cancer was diagnosed, the patient’s initial condition was not clearly immunocompromised. Preoperative evaluation and surgery were performed with an anesthesiologist, and one lung mechanical ventilator in pressure reserve volume control mode was applied during the operation [15]. After surgery, the patient was transferred to the general ward after spontaneous breathing became possible and vital signs stabilized. There were no complications such as atelectasis or fever after the operation. Steroid was administered because newly developed dyspnea and extensive GGO on chest CT and decreased SpO_2_ from 97% to 93%. Symptoms were improved. In all these diagnosis and treatment process, intensivist, pulmonologist, and thoracic surgeon participated as a team. One study has suggested that surgical treatment can be a risk factor for PCP [6]. They suggested that clinicians should not rule out the treatment of putative patients infected by *P. jirovecii,* and should also consider prophylaxis on a case-by-case basis [11]. Therefore, it is necessary to consider additional risk factors for PCP and the timing of preventive treatment. In addition, considering the high PPV of PCR results, tests should be performed in patients with suspected diseases.

## 4. Conclusions

PCP, caused by *P. jirovecii*, is a common opportunistic infection in immunocompromised individuals and is associated with high mortality rates [1,2]. In patients with no risk factors for PCP, when acute respiratory failure occurs, other causes of atypical pneumonia and COVID-19 infection should be excluded and the possibility of PCP should be considered. We report a unique case of PCP in a non-immunocompromised patient with lung cancer after surgical treatment alone. Thus, it is necessary to consider additional risk factors for PCP and the timing of preventive treatment.

## Data Availability

Not applicable.
